# Burnout among Polish paramedics: insights from the Oldenburg Burnout Inventory

**DOI:** 10.3389/fpubh.2024.1444833

**Published:** 2024-08-06

**Authors:** Justyna Kosydar-Bochenek, Dorota Religa, Kamila Iwanicka, Mateusz Szczupak, Sabina Krupa-Nurcek

**Affiliations:** ^1^Institute of Health Sciences, College of Medical Sciences of the University of Rzeszów, Rzeszów, Poland; ^2^Deputy Head of Division for Clinical Geriatrics, Department of Neurobiology, Care Sciences and Society (NVS), Karolinska Institute, Stockholm, Sweden; ^3^Students of the Innovative Research in Emergency Medicine Student Club at the University of Rzeszów, Rzeszów, Poland; ^4^Department of Anesthesiology and Intensive Care, Copernicus Hospital, Gdańsk, Poland; ^5^Department of Surgery, Institute of Medical Sciences, Medical College of Rzeszów University, Rzeszów, Poland

**Keywords:** job burnout, paramedic, emergency medical services, the Oldenburg Burnout Inventory (OLBI), occupational health

## Abstract

**Introduction:**

Emergency medical services rely heavily on paramedics who, as frontline responders, face unique stressors that can potentially lead to burnout. This pilot study utilizes the Oldenburg Burnout Inventory (OLBI) to assess burnout levels among Polish paramedics. The aim is to contribute to the understanding of burnout in this specific professional context and identify key factors influencing burnout dimensions. Future research will build on these preliminary findings.

**Materials and methods:**

A cross-sectional study was conducted from March 01 to April 30, 2023, utilizing an online survey accessible to Polish paramedics. The OLBI, a validated tool, was employed to measure burnout, focusing on two dimensions: exhaustion and withdrawal of involvement.

**Results:**

Among the 147 participating paramedics, the majority were male (65.99%). Paramedics exhibited burnout symptoms across both dimensions measured by The Oldenburg Burnout Inventory scale (OLBI), with an average level for lack of commitment recorded at 20.09, an average level for exhaustion at 20.60. The study revealed that 41.5% of paramedics experienced low burnout, 44.9% reported moderate burnout, and 13.6% faced high burnout risks. Analysis showed that women experienced significantly higher levels of exhaustion compared to men (*p* = 0.01).

**Conclusion:**

This pilot study provides valuable initial insights into burnout among Polish paramedics. The OLBI’s two-factor structure, evaluating exhaustion and disengagement, proved reliable and valid in this context. The prevalence of burnout, with over 60% of paramedics experiencing moderate to high levels, highlights the urgency of addressing burnout in this profession. Future research will be essential to explore the underlying causes and develop targeted interventions.

**Practical implications:**

Understanding the factors contributing to burnout among paramedics is crucial for developing targeted interventions. Strategies should focus on stress management training, organizational support, and well-being initiatives. Addressing gender-specific differences in burnout experiences is essential for tailoring interventions effectively. Proactive psychological support mechanisms and optimized working conditions can enhance paramedics’ overall well-being, ensuring their continued effectiveness in providing emergency medical services.

## 1 Introduction

Emergency medical services play a vital role in healthcare, with paramedics often serving as the frontline responders in critical situations. The demanding nature of their work, characterized by rapid decision-making and exposure to traumatic events, places paramedics at an increased risk of experiencing burnout. Recognizing the importance of addressing this issue, our study focuses on assessing the level of burnout among Polish paramedics, utilizing The Oldenburg Burnout Inventory (OLBI) as a comprehensive tool for measurement (1, 2).

In recent years, the prevalence of burnout among healthcare professionals, including paramedics, has garnered increased attention due to its profound impact on both individual well-being and the effectiveness of emergency medical services (3). The unique challenges faced by paramedics, such as high-stress environments, unpredictable situations, and limited control over outcomes, necessitate a targeted investigation into the specific dimensions of burnout within this professional group.

The objective of this study is to contribute to the understanding of burnout among paramedics in Poland by employing the OLBI. By focusing on this specific occupational group and utilizing a validated measurement tool, our research aims to provide nuanced insights into the prevalence and nature of burnout among Polish paramedics.

OLBI, developed by Demerouti et al. (1, 2), assesses burnout based on exhaustion and disengagement. Exhaustion refers to the feeling of emotional and physical exhaustion that paramedics often experience due to everyday difficult and emotionally taxing situations. Disengagement includes a feeling of detachment and distance from work, potentially manifesting as cynicism or decreased engagement with patients and colleagues.

Through the analysis of OLBI scores, our further studies will try seeks to identify key factors contributing to burnout, both at the individual and organizational levels. The findings hold the potential to inform targeted interventions and support mechanisms aimed at mitigating burnout among paramedics, ultimately enhancing the overall well-being of these essential healthcare professionals.

Research on paramedic burnout indicates its prevalence and impact on both individuals and organizations. A study by Alexander and Klein (4) found that nearly half of the participating paramedics reported high levels of emotional exhaustion and depersonalization, indicating burnout. High levels of burnout in paramedics have been associated with decreased job satisfaction, intention to leave the profession, and reduced quality of patient care (3).

Several factors contribute to burnout among paramedics. The demanding nature of emergency medical services, exposure to trauma and death, and high workload are key stressors. Lack of control, inadequate social support, and limited resources can further exacerbate burnout (5). Individual factors such as coping strategies, resilience, and work-life balance also play a role in determining vulnerability to burnout.

Addressing paramedic burnout requires a comprehensive approach. Organizational interventions should focus on improving working conditions, providing access to support services, implementing effective shift scheduling and rest periods, and fostering a culture of open communication and support. Halpern et al. (5) suggest that providing opportunities for debriefing and peer support can be beneficial in mitigating burnout among paramedics.

Individual-level strategies are equally important in managing burnout. Developing effective coping mechanisms, engaging in self-care practices, seeking social support, and maintaining a healthy work-life balance are crucial for paramedics to protect their well-being and resilience.

In conclusion, job burnout poses a significant challenge in the paramedic profession, impacting both paramedics and the quality of patient care. Utilizing scales such as the OLBI, we can gain insights into the experiences of paramedics and identify areas for intervention. By addressing organizational factors and promoting individual well-being, it is possible to mitigate burnout and create a healthier work environment for paramedics.

## 2 Materials and methods

### 2.1 Study design

A cross-sectional pilot study was conducted to assess burnout levels among paramedics in Poland using the Oldenburg Burnout Inventory (OLBI). The aim of this study was to provide preliminary insights into burnout within this professional context and to identify key factors influencing burnout dimensions.

### 2.2 Characteristics of the research tool

Our questionnaire contained socio-demographic information and burnout assessment, which was performed using the Oldenburg Burnout Inventory (OLBI) The original version of the Oldenburg Burnout Inventory (OLBI) was developed by Demerouti in 1999 (1).

The OLBI questionnaire contains 16 test questions, eight items to measure each dimension, each with a choice of one of four items (two of a positive nature and two of a negative nature). The premise behind constructing the questionnaire was to measure two dimensions, namely energy and identification, for a comprehensive interpretation of the results. The initial subscale is labeled as exhaustion, while the second is identified as withdrawal of involvement (disengagement) (1, 6). Validation efforts conducted by researchers, encompassing Dutch healthcare professionals and administrative staff (specifically air traffic controllers), substantiated the tool’s reliability, with Cronbach’s alpha registering at 0.85 for both dimensions. The bipartite structure of the instrument was consistently affirmed within both cohorts of participants. Moreover, the questionnaire exhibited discernment in capturing variations across professions; notably, healthcare professionals manifested a heightened susceptibility to burnout compared to their counterparts in air traffic control (1). This reliability has been corroborated across diverse international settings (6). In alignment with the authors’ recommendations, akin to the approach employed with the Maslach Burnout Inventory (MBI), an aggregate burnout score is not computed. Instead, the scores for each subscale are independently interpreted (1).

Respondents provide answers on a 4-point scale, where 1 corresponds to “agree” and 4 to “disagree.” Within each subscale, half of the items are framed negatively, and the other half positively. To ensure the unidirectionality of the scale, negatively worded items undergo inversion (i.e., the scale is reversed). The subscale scores for exhaustion and detachment from work are derived by summing the item scores and dividing by their number, resulting in scores within the range of 1 to 4. A higher score indicates elevated levels of both burnout components—exhaustion and detachment from work (1, 7, 8).

The results obtained indicated that the original version of OLBI exhibits favorable psychometric parameters, demonstrated good internal consistency and validity. The Polish version of the questionnaire has also good psychometric properties (7, 8).

The OLBI has been shown to be a reliable and valid measure of burnout. It has been used to study burnout in a variety of settings, including healthcare, education, and business. The OLBI stands out as a reliable and valid measure of burnout, offering simplicity in administration and scoring. Its extensive translation into over 20 languages ensures accessibility across diverse linguistic and cultural contexts. This tool proves versatile, allowing assessment at both individual and group levels, while its capability to track changes over time adds valuable insights into the evolving nature of burnout (9, 10).

### 2.3 Data collection, setting, and procedure

Data were gathered during the period spanning March 01 to April 30, 2023, utilizing an online survey accessible to participants. Permission to employ the survey tool was secured from its developers prior to its administration. The survey targeted paramedics within the Polish emergency medical services system. Distribution of the questionnaire occurred through the platforms of paramedic associations and via email to their membership. Additionally, the survey link was disseminated on the social media profiles of relevant organizations and shared within paramedic-centric groups. Employing an online survey method proved expeditious, straightforward, and pragmatic. The survey was conducted in the Polish language.

Prior to survey completion, respondents were provided with a concise overview of the survey’s objectives. Following the completion of the survey, participants were asked to provide brief demographic information. To ensure confidentiality, no personally identifiable details were requested from respondents. Participants affirmed their informed consent to participate in the study through questionnaire completion, with the option to withdraw from participation at any juncture. Respondents were unequivocally informed of the voluntary and anonymous nature of their involvement in the study. The entirety of the questionnaire demanded approximately 10 min for completion, indicating it was an effective measurement for paramedics due to busy work.

### 2.4 Inclusion and exclusion criteria

#### 2.4.1 Inclusion criteria

Individuals working as paramedics in emergency medical services; working in emergency care for at least 4 weeks, professionally active.

#### 2.4.2 Exclusion criteria

Persons who work as paramedics or less than 4 weeks; other medical personnel, with no professional activity.

### 2.5 Ethical considerations

The University of Rzeszow’s Bioethics Committee gave its approval to this study (KBE No. 2022/013). The authors adhered to the Declaration of Helsinki’s guidelines (World Medical Association, 2013).

### 2.6 Statistics

Statistical analyses were conducted using Statistica software (v13.3, StatSoft, Poland). For quantitative data, the presentation included the mean and standard deviation (SD). The choice of statistical tests depended on both the number of groups compared and the results of the Shapiro–Wilk test. Statistical significance was determined at a threshold of *p* < 0.05.

## 3 Results

### 3.1 Participants

A total of 147 participants contributed to the study, comprising 50 women (34.01%) and 97 men (65.99%). The age range of survey participants spanned from 22 to over 55 years. Among the surveyed individuals, a majority resided in urban areas, constituting 72.11% of the respondents. Regarding educational attainment, those holding a bachelor’s degree constituted the majority at 66.67%, followed by respondents with a master’s degree at 24.49%, the overwhelming majority of rescuers perform their duties in pre-hospital conditions and are employed in emergency medical teams (58.51%). Paramedics with 6–10 years of experience constituted the largest group (46.84%), with a large group also consisting of people with less than 5 years of experience (32.55%). Table 1 details the characteristics of the study participants.

**Table 1:** Participants’ characteristics (*N* = 147).

		*N*	%
1.	*Gender*		
	Female	50	34.01%
	Male	97	65.99%
2.	*Age*		
	22–25	44	29.93%
	26–35	57	38.78%
	35–45	30	20.41%
	>45	16	10.88%
3.	*Residence area*		
	Urban area	106	72.11%
	Rural area	41	27.89%
4.	*Education level*		
	Master’s degree	36	24.49%
	Bachelor degree	98	66.67%
	Postsecondary	13	8.84%
5.	*Workplace*		
	Pre-hospital EMS	86	58.51%
	Emergency department (ED)	22	14.96%
	Both EMS and ED	39	26.53%
6.	*Total experience in work*		
	≤5 years	38	25.85%
	6–10 years	103	70.07%
	11–20 years	5	3.40%
	≥20 years	1	0.68%

### 3.2 Results of the Oldenburg Burnout Inventory in the research group

The average OLBI scores were 20.09 and 20.60 for disengagement and exhaustion, respectively (Table 2).

**Table 2:** The OLBI questionnaire score in the study group (*N* = 147).

	*x*	Me	Min	Max	SD
Exhaustion	20.60	21	8	32	4.56
Disengagement	20.09	20	9	32	4.23

**Figure 1:**
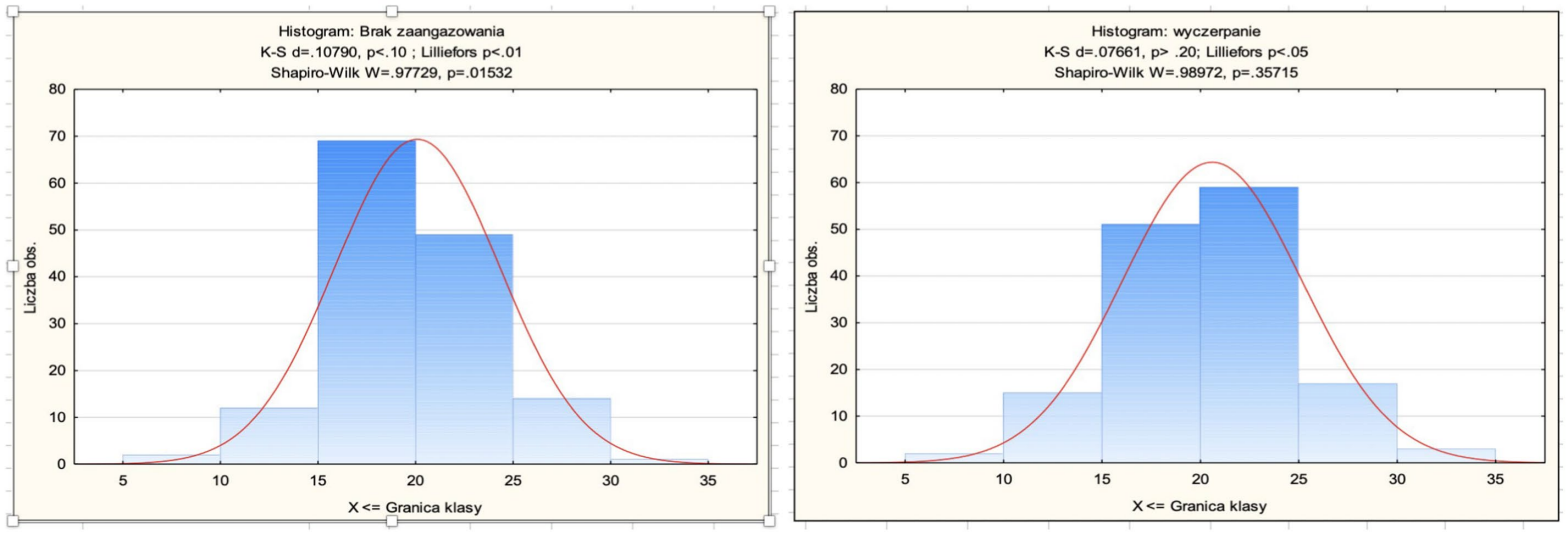
Histogram of disengagement and histogram of exhaustion.

Figure 1 shows the detailed distribution of results for both variables: disengagement and exhaustion.

The results of the study confirmed that the OLBI questionnaire has good psychometric properties and is a reliable tool for measuring burnout. The Cronbach’s alpha coefficient pointed out a value of 0.79 for disengagement and 0.86 for exhaustion and the factorial analysis revealed that the two components, exhaustion and disengagement, loaded on one factor, burnout (Table 3).

**Table 3:** Psychometric properties of the OLBI questionnaire based on own study (*N* = 147).

Burnout component	Number of items	Number of cases	Cronbach’s alpha	Average correlation	Number of items
Disengagement	8	147	0.79	0.33	8
Exhaustion	8	147	0.86	0.44	8

The study group was divided according to the criteria recommended by the authors of the scale into 3 groups:

- High level: individuals who are professionally burned out (48–64 points).
- Medium level: persons who are at risk of professional burnout (40–47 points).
- Low level: persons who are not professionally burned out (less than 40 points).

**Figure 2:**
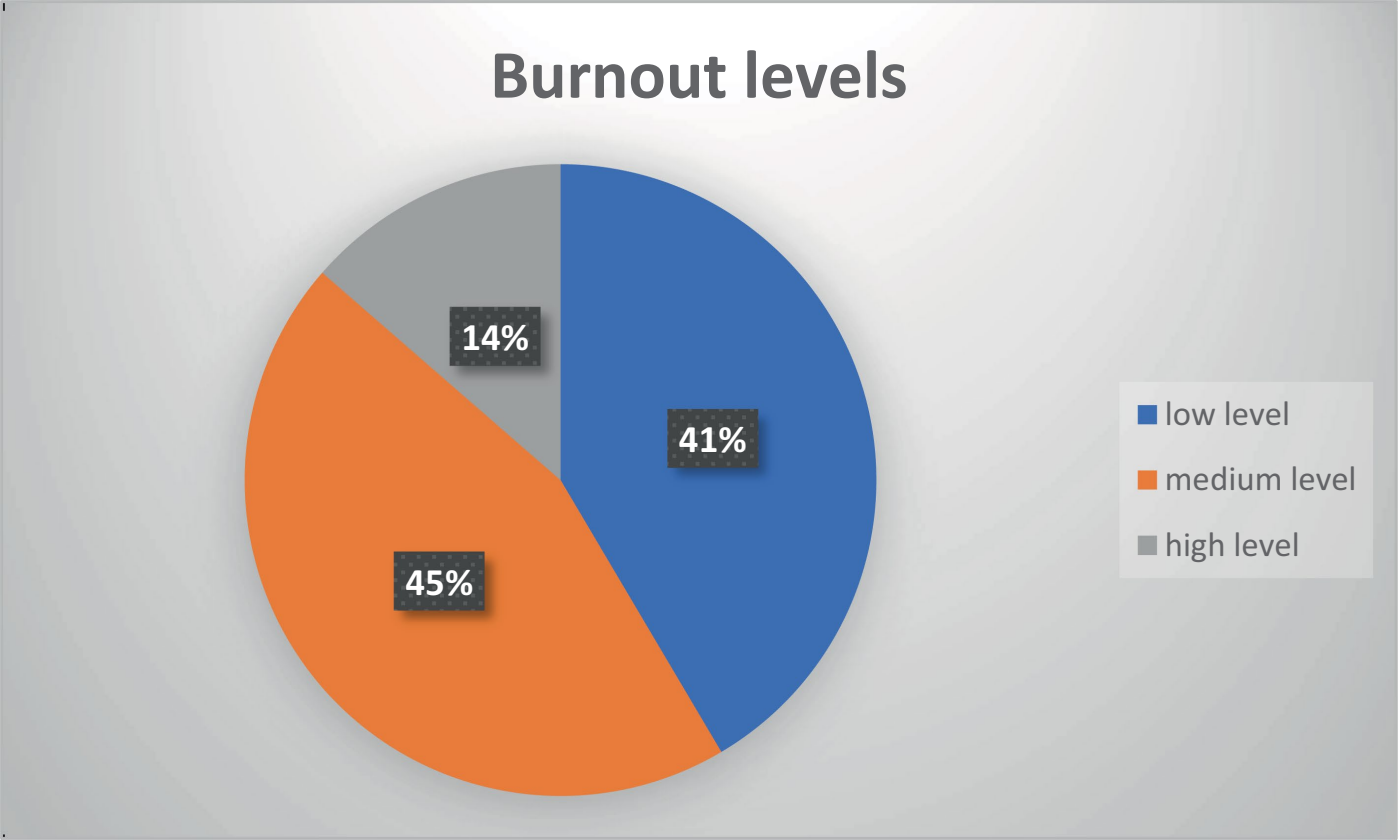
Burnout levels in the study group (*N* = 147).

Figure 2 shows how the occupational burnout index is distributed among the study group. 13.6% of the respondents, or 20 people, belongs to the high-risk group of burnouts, and can be classified as occupational burnout according to the index and methodology of the OLBI (high level). Almost half of all respondents (44.9%) belong to the group of people at risk of burnout (medium level). The remaining group, accounting for 41.5% of the respondents, are those who are not professionally burned out (low level). The group of those who are professionally burned out and those who are threatened by burnout together make up almost 60% of the respondents, a worrying result and one that requires further study.

### 3.3 The impact of socio-demographic factors on burnout

**Figure 3:**
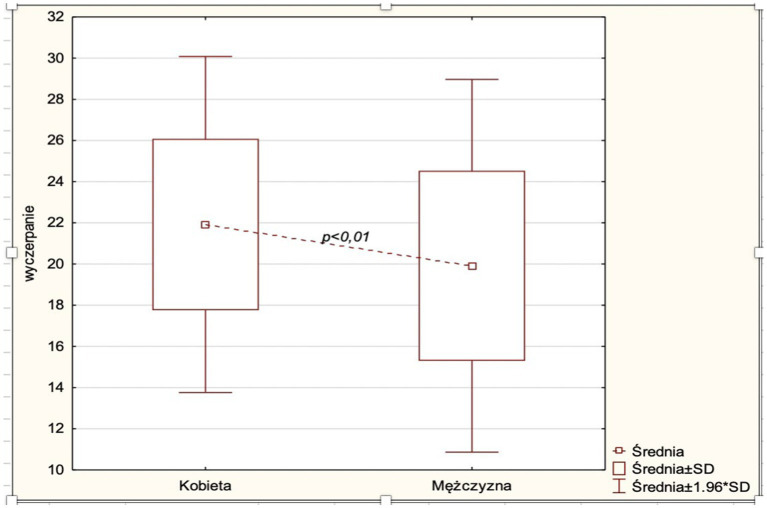
Average scores of the exhaustion variable by gender.

The study showed that women had significantly higher levels of exhaustion than men (*p* = 0.01). The average score in the area of exhaustion for women was 21.92, for men 19.92. The results are shown in Figure 3.

For the other variables, i.e., age, area of residence, education level, workplace and total experience in work, no relationship was shown.

## 4 Discussion

In research exploring occupational burnout, various factors are scrutinized, including excessive workloads, workplace violence, managerial support deficiencies, prolonged working hours, a lack of job control, and inadequate remuneration. Findings from these studies are contingent on the employed methodology, organizational culture, healthcare systems, and other country-specific influences (11–13). There is still a lack of research on the issue of burnout in the professional group of paramedics. It is imperative to acknowledge that the landscape of burnout among paramedics is dynamic and can evolve over time. Ongoing research endeavors continue to shape our understanding, and results may shift with the evolution of research and the implementation of effective management strategies. For paramedics, occupational burnout stands as a formidable threat, given the daily confrontations with highly stressful situations demanding significant expertise, knowledge, and both physical and mental aptitude. Their routine exposure to crisis scenarios, accidents, and tragedies can deeply impact their mental well-being, with frequent exposure to extreme suffering and death potentially leading to psychological exhaustion and trauma. Operating under time constraints in a challenging and often changing environment further compounds the stress. An integral but often overlooked aspect is an overburdened healthcare system that compels medical personnel, including paramedics, to extend their working hours (14). This not only becomes burdensome but generates a sense of exhaustion due to the inadequate provision of time for rest and relaxation (3, 15). Stress, trauma, and occupational burnout are increasingly prevalent issues among healthcare workers globally, extending beyond Poland to international contexts (13, 16–19).

The findings of this study echo those of a research initiative in Poland conducted by Witczak-Błoszczyk et al. (20). Notably, the investigation highlights a high incidence of professional burnout and vulnerability among medical personnel. The amalgamated results from respondents of both questionnaires unequivocally indicate a deteriorating trend in professional burnout.

Professional burnout among medical personnel poses a significant challenge confronted by emergency medical systems worldwide. The United Kingdom, for instance, grapples with this issue among ambulance workers, as evidenced by a study indicating that only 13.6% of participants met burnout criteria, while 44.9% were at risk of developing the syndrome. Because of that, over 50% of surveyed paramedics in the UK exhibited symptoms of professional burnout, underscoring its pervasive nature within the emergency medical service milieu and it’s potential to result in sickness absenteeism and compromised mental health (21).

German paramedics, contending with daily stressors in providing medical assistance, face an escalating lack of resources and heightened healthcare demands. The German emergency medical system’s emphasis on swift and effective responses places paramedics in situations where they must make critical life-and-death decisions under time constraints. The witnessing of suffering and traumatic scenes contributes to chronic stress and professional burnout. Factors identified by Natalie Baier et al. (22) in Germany include excessive workloads, insufficient support from superiors and the healthcare system, a dearth of rest opportunities, lack of recognition, and low remuneration. To address professional burnout among German paramedics, concerted efforts are required at both the emergency medical unit and healthcare system levels.

In India, another country with a healthcare system grappling with formidable challenges, paramedics share similar stressors with their Polish counterparts. High workload, inadequate resources, low remuneration, and a lack of recognition compound the challenges faced by paramedics in India. These factors contribute significantly to burnout, leading to frustration and professional exhaustion. Paramedics in India also encounter a lack of support and understanding from society, exacerbating feelings of burnout. Additionally, they grapple with traumatic situations such as accidents, infectious diseases, and natural disasters, heightening the pressure and exposing them to psychological trauma. The ramifications of professional burnout among paramedics extend beyond individual well-being, adversely impacting the quality of healthcare provided to patients. Physically and emotionally exhausted paramedics possess limited resources for aiding others, while burnout contributes to elevated rates of absenteeism and turnover, exacerbating challenges within the healthcare system (23).

The emergence of the COVID-19 pandemic has notably escalated burnout rates among medical personnel, as corroborated by research conducted in Poland, Spain, and the United States (20, 24, 25). Paramedics, serving as crucial frontline healthcare workers, have borne the brunt of challenges posed by the pandemic. Prolonged exposure to the intense nature of the pandemic has subjected paramedics to heightened workloads, with an influx of patients requiring urgent attention. The resultant long hours and limited breaks have led to chronic fatigue and stress. Witnessing the suffering and death associated with the pandemic has had a profound impact on the mental health of paramedics, contributing to feelings of helplessness, discouragement, and emotional exhaustion. Shortages in medical resources and equipment have further compounded burnout, forcing paramedics to operate without adequate protection, increasing the risk of infection. The lack of support and recognition from authorities and the public has added to the burnout challenge, leaving paramedics feeling unappreciated and ignored. These multifaceted factors collectively contribute to the surge in burnout among paramedics during the ongoing COVID-19 pandemic (26, 27).

## 5 Conclusion

Paramedics represent a professional cohort particularly susceptible to experiencing burnout. Within the surveyed group, nearly 60% of respondents either met the criteria for burnout or were at risk of developing this syndrome. Gender disparity was observed, with female paramedics demonstrating a higher likelihood of developing burnout compared to their male counterparts. Burnout poses a significant challenge, impacting work, family, and societal functioning, which can lead to reduced work efficiency and potentially compromise patient safety.

This pilot study provides initial insights into the prevalence and dimensions of burnout among Polish paramedics. Future research will be essential to further investigate these findings, particularly by exploring underlying causes and developing targeted interventions. Future studies should also address the gender disparities observed and examine the specific workplace stressors and coping mechanisms that contribute to burnout.

To mitigate burnout among paramedics, strategic measures should include mandatory training on stress reactions, workshops focusing on coping skills, and initiatives aimed at enhancing working conditions within the paramedic profession. Proactive psychological support is crucial for paramedics exhibiting symptoms of burnout, underscoring the importance of professional care to address and manage their well-being.

## 6 Implications for practice

Understanding the factors contributing to burnout among paramedics is crucial for developing effective interventions. Strategies should focus on stress management training, organizational support, and well-being initiatives. It is also important to address gender-specific differences in burnout experiences to tailor interventions effectively.

Implementing proactive psychological support mechanisms and optimizing working conditions can significantly improve paramedics’ overall well-being and enhance their effectiveness in providing emergency medical services. Integrating stress management training into the medical education curriculum can help paramedics handle work-related stress more effectively, develop personal coping techniques, and potentially prevent burnout. This approach can strengthen their resilience and improve their overall performance in delivering high-quality emergency care.

## Data availability statement

The raw data supporting the conclusions of this article will be made available by the authors, without undue reservation.

## Author contributions

JK-B: Conceptualization, Data curation, Formal analysis, Funding acquisition, Investigation, Methodology, Project administration, Resources, Software, Supervision, Validation, Visualization, Writing – original draft, Writing – review & editing. DR: Funding acquisition, Resources, Supervision, Visualization, Writing – review & editing. KI: Data curation, Investigation, Software, Writing – original draft. MS: Methodology, Supervision, Writing – review & editing. SK: Conceptualization, Investigation, Supervision, Visualization, Writing – review & editing.

## References

[1] Demerouti E Bakker AB. The Oldenburg Burnout Inventory: a good alternative to measure burnout and engagement In: Handbook of stress and burnout in health care (2008). 65–78.

[2] Demerouti E Bakker AB Nachreiner F Schaufeli WB. The job demands-resources model of burnout. J Appl Psychol. (2001) 86:499–512. doi: 10.1037/0021-9010.86.3.499 11419809

[3] Reardon M Abrahams R Thyer L Simpson P. Review article: prevalence of burnout in paramedics: a systematic review of prevalence studies. Emerg Med Australas. (2020) 32:182–9. doi: 10.1111/1742-6723.13478, PMID: 32067408

[4] Alexander DA Klein S. Ambulance personnel and critical incidents: impact of accident and emergency work on mental health and emotional well-being. Br J Psychiatry. (2001) 178:76–81. doi: 10.1192/bjp.178.1.76 11136215

[5] Crowe RP Bower JK Cash RE Panchal AR Rodriguez SA Olivo-Marston SE. Association of burnout with workforce-reducing factors among EMS professionals. Prehosp Emerg Care. (2018) 22:229–36. doi: 10.1080/10903127.2017.1356411 28841102

[6] Demerouti E Bakker AB Vardakou I Kantas A. The convergent validity of two burnout instruments: a multitrait-multimethod analysis. Eur J Psychol Assess. (2003) 19:12–23. doi: 10.1027//1015-5759.19.1.12

[7] Chirkowska-Smolak T. A Polish adaptation of the Oldenburg Burnout Inventory (OLBI). Studia Oecon Posnan. (2018) 6:24–47. doi: 10.18559/SOEP.2018.3.2

[8] Baka Ł Basińska BA. Psychometric properties of the Polish version of the Oldenburg Burnout Inventory (OLBI). Med Pr. (2016) 67:29–41. doi: 10.13075/mp.5893.00353, PMID: 27044717

[9] Tipa RO Tudose C Pucarea VL. Measuring burnout among psychiatric residents using the Oldenburg Burnout Inventory (OLBI) instrument. J Med Life. (2019) 12:354–60. doi: 10.25122/jml-2019-0089, PMID: 32025253 PMC6993305

[10] Block RI Bair HL Carillo JF. Is exhaustion more sensitive than disengagement to burnout in academic anesthesia? A study using the Oldenburg Burnout Inventory. Psychol Rep. (2020) 123:1282–96. doi: 10.1177/0033294119856560, PMID: 31219406

[11] Braun D Reifferscheid F Kerner T Dressler JL Stuhr M Wenderoth S. Association between the experience of violence and burnout among paramedics. Int Arch Occup Environ Health. (2021) 94:1559–65. doi: 10.1007/s00420-021-01693-z, PMID: 33885950

[12] Kang JH Sakong J Kim JH. Impact of violence on the burnout status of paramedics in the emergency department: a multicenter survey study. Australas Emerg Care. (2022) 25:147–53. doi: 10.1016/j.auec.2021.07.002, PMID: 34284977

[13] Grochowska A Gawron A Bodys-Cupak I. Stress-inducing factors vs. the risk of occupational burnout in the work of nurses and paramedics. Int J Environ Res Public Health. (2022) 19:5539. doi: 10.3390/ijerph19095539, PMID: 35564934 PMC9104409

[14] Demerouti E Mostert K Bakker AB. Burnout and work engagement: a thorough investigation of the independency of both constructs. J Occup Health Psychol. (2010) 15:209–22. doi: 10.1037/a0019408, PMID: 20604629

[15] Patterson PD Buysse DJ Weaver MD Callaway CW Yealy DM. Recovery between work shifts among emergency medical services clinicians. Prehosp Emerg Care. (2015) 19:365–75. doi: 10.3109/10903127.2014.995847, PMID: 25658148

[16] Sahebi A Golitaleb M Jahangiri K. Occupational burnout in pre-hospital emergency personnel in Iran: a systematic review and meta-analysis. Iran J Nurs Midwifery Res. (2021) 26:11–7. doi: 10.4103/ijnmr.IJNMR_175_20, PMID: 33954093 PMC8074727

[17] Xu H Yuan Y Gong W Zhang J Liu X Zhu P. Reliability and validity of the Chinese version of Oldenburg Burnout Inventory for Chinese nurses. Nurs Open. (2022) 9:320–8. doi: 10.1002/nop2.1065, PMID: 34546665 PMC8685855

[18] Sinval J Queirós C Pasian S Marôco J. Transcultural adaptation of the Oldenburg Burnout Inventory (OLBI) for Brazil and Portugal. Front Psychol. (2019) 10:338. doi: 10.3389/fpsyg.2019.00338, PMID: 30914985 PMC6422925

[19] Nirel N Goldwag R Feigenberg Z Abadi D Halpern P. Stress, work overload, burnout, and satisfaction among paramedics in Israel. Prehosp Disaster Med. (2008) 23:537–46. doi: 10.1017/s1049023x00006385, PMID: 19557971

[20] Witczak-Błoszyk K Krysińska K Andriessen K Andriessen K Stańdo J Czabański A. Work-related suicide exposure, occupational burnout, and coping in emergency medical services personnel in Poland. Int J Environ Res Public Health. (2022) 19:1156. doi: 10.3390/ijerph19031156 35162179 PMC8835152

[21] Miller E. The prevalence of stress and burnout in UK emergency ambulance service workers and its impact on their mental health and well-being. Br Paramed J. (2021) 5:62–3. doi: 10.29045/14784726.2021.3.5.4.62, PMID: 34421379 PMC8341057

[22] Baier N Roth K Felgner S Henschke C. Burnout and safety outcomes—a cross-sectional nationwide survey of EMS-workers in Germany. BMC Emerg Med. (2018) 18:24. doi: 10.1186/s12873-018-0177-2, PMID: 30126358 PMC6102842

[23] Baruah A Das S Dutta A Das B Sharma T Hazarika M. Degree and factors of burnout among emergency healthcare workers in India. Int J Sci Res. (2019) 8:41–5. doi: 10.15373/22778179, PMID: 31069180 PMC6502256

[24] Amro TM Arcos GP Montero VE Castro DR. Impact of COVID-19 pandemic on stress and burnout levels amongst emergency medical technicians: a cross-sectional study in Spain. Ann Med. (2022) 54:3007–16. doi: 10.1080/07853890.2022.2137735, PMID: 36314513 PMC9629066

[25] Firew T Sano ED Lee JW Flores S Lang K Salman K. Protecting the front line: a cross-sectional survey analysis of the occupational factors contributing to healthcare workers’ infection and psychological distress during the COVID-19 pandemic in the USA. BMJ Open. (2020) 10:e042752. doi: 10.1136/bmjopen-2020-042752, PMID: 33087382 PMC7580061

[26] Lai J Ma S Wang Y Cai Z Hu J Wei N. Factors associated with mental health outcomes among health care workers exposed to coronavirus disease 2019. JAMA Netw Open. (2020) 3:e203976. doi: 10.1001/jamanetworkopen.2020.3976, PMID: 32202646 PMC7090843

[27] Koontalay A Suksatan W Prabsangob K Sadang JM. Healthcare workers’ burdens during the COVID-19 pandemic: a qualitative systematic review. J Multidiscip Healthc. (2021) 14:3015–25. doi: 10.2147/JMDH.S330041, PMID: 34737573 PMC8558429

